# Paclitaxel liposome (Lipusu) based chemotherapy combined with immunotherapy for advanced non-small cell lung cancer: a multicenter, retrospective real-world study

**DOI:** 10.1186/s12885-024-11860-3

**Published:** 2024-01-18

**Authors:** Ran Li, Hongge Liang, Jun Li, Zhenyu Shao, Donghong Yang, Jing Bao, Keqiang Wang, Wen Xi, Zhancheng Gao, Renhua Guo, Xinlin Mu

**Affiliations:** 1 Department of Respiratory and Critical Care Medicine, Lung Cancer Center, Peking University People’s Hospital, No.11 Xizhimen South Street, Xicheng District, 100044 Beijing, China; 2 Department of Oncology, The First Affiliated Hospital of Nanjing Medical University, 210029 Nanjing, China; 3 Department of Radiation Oncology, Qilu Hospital of Shandong University, 250012 Jinan, China

**Keywords:** Paclitaxel liposome, Immunotherapy, Immune checkpoint inhibitor, Non-small cell lung cancer

## Abstract

**Background:**

Paclitaxel liposome (Lipusu) is known to be effective in non-small cell lung cancer (NSCLC) as first-line treatment. This study aimed to evaluate the effectiveness and safety of paclitaxel liposome based chemotherapy plus PD-1/PD-L1 inhibitor in patients with advanced NSCLC.

**Methods:**

In this multicenter, retrospective, real-world study, patients with advanced NSCLC who were administered paclitaxel liposome based chemotherapy plus PD-1/PD-L1 inhibitor in three centers (Peking University People’s Hospital as the lead center) in China between 2016 and 2022 were included. Progression-free survival (PFS), overall survival (OS), objective response rate, disease control rate, and adverse events (AEs) were evaluated.

**Results:**

A total of 49 patients were included, with 33 (67.3%) receiving paclitaxel liposome based chemotherapy plus PD-1/PD-L1 inhibitor as first-line treatment. There were 34 patients (69.4%) diagnosed with squamous cell carcinoma and 15 (30.6%) with adenocarcinoma. The median follow-up was 20.5 (range: 3.1–41.1) months. The median PFS and OS of all patients were 9.7 months (95% confidence interval [CI], 7.0-12.4) and 30.5 months (95% CI, not evaluable-not evaluable), respectively. Patients with squamous cell carcinoma and adenocarcinoma had median PFS of 11 months (95%CI, 6.5–15.5) and 9.3 months (95%CI, 7.0-12.4), respectively. The median PFS was 9.9 months (95%CI, 7.1–12.7) in patients who received the combined regimen as first-line treatment. Treatment-related AEs of any grade were observed in 25 (51.0%) patients, and AEs of grade 3 or worse were observed in nine patients (18.4%). The most common treatment-related AEs were myelosuppression (14.3%) and fever (10.2%).

**Conclusions:**

Paclitaxel liposome based chemotherapy plus PD-1/PD-L1 inhibitor prolonged the PFS in advanced NSCLC with acceptable safety, which was worthy of clinical application.

## Background

Non-small cell lung cancer (NSCLC) is the leading cause of malignancy-related death and accounts for more than 80% of all lung cancer cases [1]. The most common histologic types of NSCLC are adenocarcinoma and squamous cell carcinoma [2]. More than 60% of patients were diagnosed at stages III and IV [3]. In the past, the combination chemotherapy was considered the standard first-line treatment for patients without driver gene mutations [4]. However, the associated benefit was quite limited, with a median overall survival (OS) of about 1 year [5].

Immune checkpoint inhibitors (ICIs), combined with chemotherapy, can be beneficial for patients with advanced NSCLC, including squamous cell carcinoma and adenocarcinoma [6, 7]. The KEYNOTE-407 study demonstrated that compared with chemotherapy alone, pembrolizumab combined with paclitaxel or nab-paclitaxel plus platinum significantly prolonged OS in untreated patients with advanced squamous NSCLC [8]. Besides, the efficacy of atezolizumab [9], tislelizumab [10], sintilimab [11], and camrelizumab [12] in combination with chemotherapy in the first-line treatment of advanced squamous NSCLC have been demonstrated. On the other hand, the KEYNOTE 021G and KEYNOTE 189 studies showed that pembrolizumab plus chemotherapy conferred benefits to patients with non-squamous NSCLC [13, 14]. The IMpower 130 and Impower 150 studies showed that atezolizumab plus chemotherapy with or without bevacizumab significantly improved the OS of patients with non-squamous NSCLC [15, 16]. Thus, based on extensive clinical evidence, chemotherapy combined with immunotherapy has become the standard first-line treatment for patients without driver gene mutations, as recommended by the NCCN guidelines (version 5.2023) [17].

Paclitaxel liposome is paclitaxel encapsulated by liposomes made from lecithin and cholesterol [18]. Paclitaxel liposome reduces the incidence of drug-induced toxicity compared with paclitaxel because polyethoxylated castor oil is not used in paclitaxel liposome, which reduces hypersensitivity reactions and peripheral neuropathy [19–21]. Additionally, paclitaxel liposome increases drug exposure by maintaining high drug concentrations in tumor tissues. Furthermore, it can treat lymphatic metastases by targeting lymph nodes because of its slow clearance through lymphatic vessels and lymph nodes [18]. Lipusu, the first commercially available form of paclitaxel liposome approved in 2003, is widely used to treat solid tumors, including NSCLC [22, 23]. In a multicenter randomized controlled trial, paclitaxel liposome plus cisplatin had comparable median progression-free survival (PFS, 5.2 months vs. 5.5 months) and median OS (14.6 months vs. 12.5 months) to gemcitabine plus cisplatin in the first-line treatment of lung squamous cell carcinoma, with significantly reduced toxicities [24]. Nevertheless, paclitaxel liposome combined with immunotherapy has not been assessed in patients with NSCLC so far.

Therefore, we conducted a multicenter, retrospective, real-world study to evaluate the effectiveness and safety of paclitaxel liposome based chemotherapy combined with PD-1/PD-L1 inhibitor in the treatment of patients with advanced NSCLC.

## Methods

### Study design and patients

In this multicenter, retrospective, real-world study, patients with advanced NSCLC in three centers in China from 2016 to 2022 were included. Inclusion criteria were: (1) histologically or cytologically confirmed stage III-V NSCLC; (2) Eastern Cooperative Oncology Group performance status (ECOG PS) score of 0–1; (3) at least one measurable lesion according to the Response Evaluation Criteria in Solid Tumors (RECIST) version 1.1; and (4) complete data of diagnosis and treatment. Patients combined with other malignant tumors were excluded. This retrospective study was approved by the Ethics Committee of the Peking University People’s Hospital (approval number 2022PHB266). Informed consent was waived by the Ethics Committee of the Peking University People’s Hospital due to the retrospective nature of this study. All procedures performed in studies involving human participants were in accordance with the ethical standards of the institutional and/or national research committee and with the 1964 Helsinki Declaration and its later amendments or comparable ethical standards.

### Treatment

Patients receiving paclitaxel liposome based chemotherapy in combination with PD-1/PD-L1 inhibitors were included, regardless of the number of treatment lines and whether targeted agents were used. The dose of the PD-1/PD-L1 inhibitor was in accordance with instructions, and it could also be used as maintenance therapy. The paclitaxel liposome dose was based on the instructions and the adjustments made according to the clinician’s experience and the patient’s tolerance were recorded.

### Outcomes

PFS was defined as the time from treatment initiation to disease progression or death from any cause. The objective response rate (ORR) was defined as the proportion of patients who achieved complete response (CR) or partial response (PR) according to RECIST version 1.1. The disease control rate (DCR) was defined as the proportion of patients who achieved CR, PR, or stable disease (SD). OS was defined as the time from treatment initiation to death from any cause.

For safety assessment, adverse events (AEs) were coded using the medical dictionary for regulatory activities (MedDRA, version 14.1), and AEs were recorded and graded according to the National Cancer Institute Common Terminology Criteria for Adverse Events (CTCAE).

### Statistical analysis

IBM SPSS (version 26.0; IBM Corp., Armonk, NY, USA) was used for all statistical analyses. Quantitative variables with normal and non-normal distributions were described as mean ± standard deviation and median (interquartile range [IQR]), respectively. Categorical variables were presented as numbers and percentages. Median OS and PFS and their 95% confidence intervals (CIs) were estimated by the Kaplan-Meier method.

## Results

### Baseline characteristics of patients

A total of 49 patients with NSCLC who received paclitaxel liposome based chemotherapy plus immunotherapy between 2016 and 2022 were included, with an average age of 65.3 ± 8.9 years. There were 40 (81.6%) males and 9 (18.4%) females. Regarding the pathological type, 34 patients (69.4%) were diagnosed with squamous cell carcinoma and 15 (30.6%) with adenocarcinoma. Twenty-eight (57.1%) patients received no prior treatments, including surgical resection or radiotherapy (Table 1). The median follow-up was 20.5 (range: 3.1–41.1) months.

**Table 1:** Baseline characteristics of patients

Variables	All (*n* = 49)
Age (years), mean ± SD	65.3 ± 8.9
Sex, n (%)	
Male	40 (81.6%)
Female	9 (18.4%)
Pathological type, n (%)	
Squamous cell carcinoma	34 (69.4%)
Adenocarcinoma	15 (30.6%)
Clinical stage, n (%)	
III	22 (44.9%)
IV	26 (53.1%)
NA	1 (2.0%)
EGFR mutations, n (%)	
Positive	3 (6.1%)
Negative	16 (32.7)
NA	30 (61.2%)
PD-L1 (TPS), n (%)	
< 1%	2 (4.1%)
1-49%	2 (4.1%)
≥ 50%	5 (10.2%)
NA	40 (81.6%)
Previous treatment, n (%)	
Surgery	2 (4.1%)
Surgery plus chemotherapy/targeted therapy	2 (4.1%)
Surgery plus radiotherapy	1 (2.0%)
Radiotherapy plus chemotherapy/target therapy	2 (4.1%)
Without prior treatment	28 (57.1%)
Previous medications, n (%)	
Pemetrexed	11 (22.4%)
Cisplatin	10 (20.4%)
Carboplatin	7 (14.3%)
Gefitinib	5 (10.2%)
Bevacizumab	4 (8.2%)
Gemcitabine	3 (6.1%)
Osimertinib	3 (6.1%)
Paclitaxel	3 (6.1%)
Endostar	3 (6.1%)
Toripalimab	2 (4.1%)
Nedaplatin	2 (4.1%)
Anlotinib	2 (4.1%)
Docetaxel	2 (4.1%)
Sintilimab	1 (2.0%)
Pembrolizumab	1 (2.0%)
Nivolumab	1 (2.0%)
Paclitaxel liposome	1 (2.0%)
Vinorelbine	1 (2.0%)

SD, standard deviation; NA, not applicable

### Treatment patterns

Thirty-three patients (67.3%) received paclitaxel liposome based chemotherapy combined with PD-1/PD-L1 inhibitor in the first-line setting. The median dose of paclitaxel liposome was 135.0 mg/m^2^, and the median total dose of paclitaxel liposome was 240.0 mg. The median number of cycles of paclitaxel liposome based chemotherapy was 5.0 (3.0, 6.0) (Table 2). For the combined immunotherapy, the most common drugs were camrelizumab (32.7%) and tislelizumab (20.4%). For combined chemotherapy or targeted therapy, the most common drugs were carboplatin (38.8%) and nedaplatin (20.4%) (Table 3). Of the 16 patients who were administered maintenance therapy, five received 7–9 cycles of paclitaxel liposome combined with immunotherapy as maintenance therapy, while the others received immunotherapy alone.

**Table 2:** Applications of paclitaxel liposome

Variables	All (*n* = 49)
Treatment line of paclitaxel liposome, n (%)	
First-line	33 (67.3%)
Second-line	9 (18.4%)
Third-line	1 (2.0%)
Fourth-line	3 (6.1%)
Fifth-line	3 (6.1%)
Dose of paclitaxel liposome (mg/m^2^), median (IQR)	135.0 (127.0, 150.0)
Total dose of paclitaxel liposome (mg), median (IQR)	240.0 (210.0, 270.0)
Cycles of paclitaxel liposome therapy, median (IQR)	5.0 (3.0, 6.0)
Cycles of paclitaxel liposome therapy, n (%)	
1	2 (4.1%)
2	9 (18.4%)
3	6 (12.2%)
4	6 (12.2%)
5	9 (18.4%)
6	5 (10.2%)
7	2 (4.1%)
8	3 (6.1%)
9	1 (2.0%)
10	1 (2.0%)
13	1 (2.0%)
14	1 (2.0%)
15	3 (6.1%)

IQR, interquartile range

**Table 3:** Combination regimen

Regimens	All (*n* = 49)
Immunotherapy, n (%)	
Camrelizumab	16 (32.7%)
Tislelizumab	10 (20.4%)
Pembrolizumab	8 (16.3%)
Sintilimab	6 (12.2%)
Toripalimab	5 (10.2%)
Nivolumab	4 (8.2%)
Durvalumab	1 (2.0%)
Atezolizumab	1 (2.0%)
Chemotherapy or targeted therapy, n (%)	
Carboplatin	19 (38.8%)
Nedaplatin	10 (20.4%)
Cisplatin	3 (6.1%)
Anlotinib	3 (6.1%)
Lobaplatin	3 (6.1%)
Apatinib	1 (2.0%)
Endostar	1 (2.0%)

### Effectiveness

**Fig. 1:**
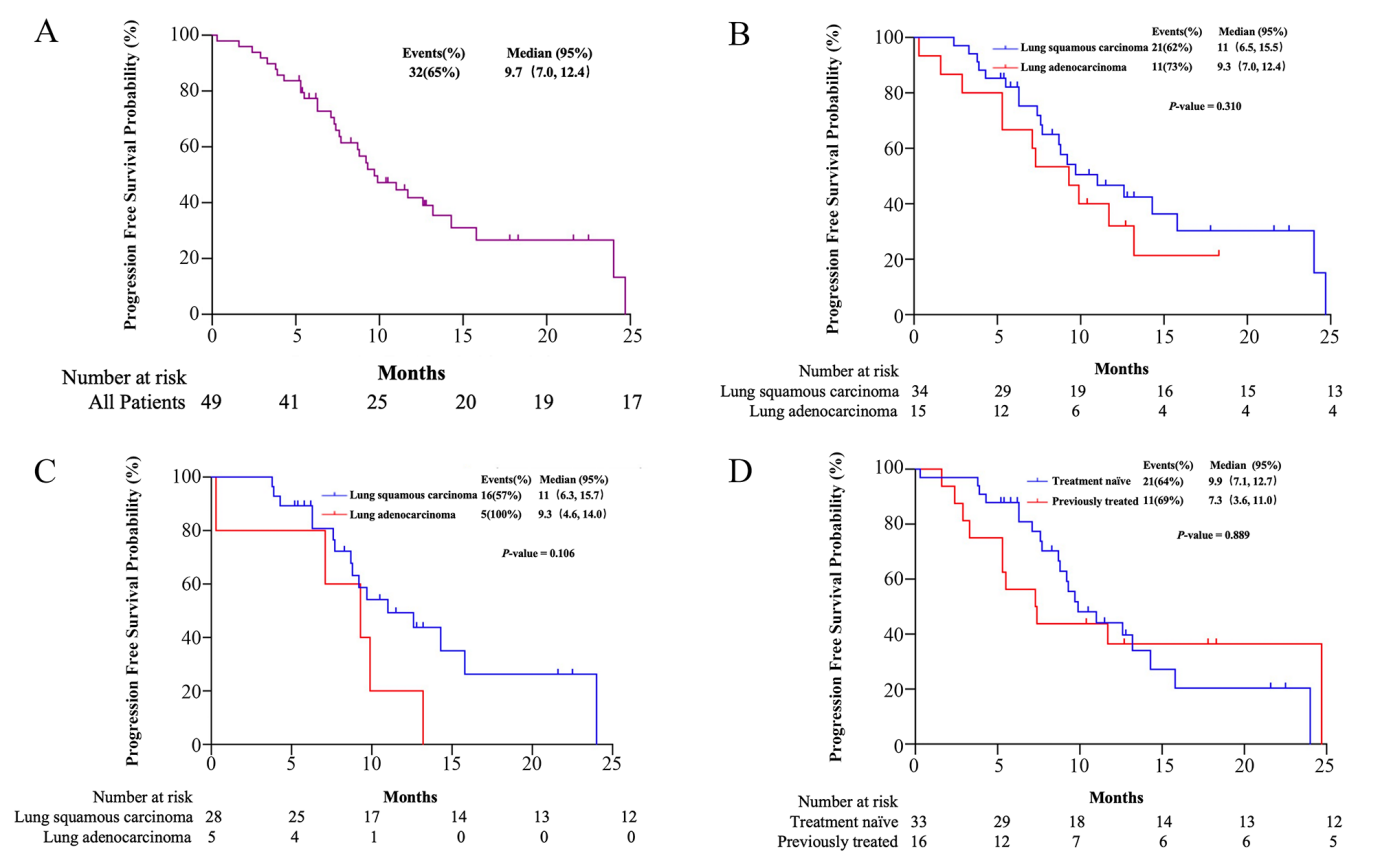
Kaplan-Meier curves for progression-free survival in all patients (**A**), patients with squamous cell carcinoma and adenocarcinoma (**B**), patients with squamous cell carcinoma and adenocarcinoma who received paclitaxel liposome based chemotherapy plus immunotherapy as first-line therapy (**C**), and patients who received paclitaxel liposome based chemotherapy plus immunotherapy as first-line therapy, and second- or later-line treatment (**D**)

The median PFS in all patients was 9.7 months (95% CI, 7.0-12.4) (Fig. 1A). Patients with squamous cell carcinoma and adenocarcinoma had median PFS of 11 months (95% CI, 6.5–15.5) and 9.3 months (95% CI, 7.0-12.4), respectively (Fig. 1B). In patients with squamous cell carcinoma and adenocarcinoma who were administered paclitaxel liposome based chemotherapy plus PD-1/PD-L1 inhibitor as first-line therapy, median PFS were 11 months (95% CI, 6.3–15.7) and 9.3 months (95% CI, 4.6–14.0), respectively (Fig. 1C). The median PFS was 9.9 months (95% CI, 7.1–12.7) in patients administered paclitaxel liposome based chemotherapy plus PD-1/PD-L1 inhibitor in the first-line setting, and 7.3 months (95% CI, 3.6–11.0) in the second- or later-line setting (Fig. 1D).

**Fig. 2:**
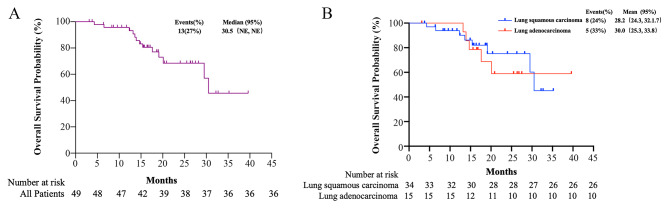
Kaplan-Meier curves for overall survival in all patients (**A**), and patients with squamous cell carcinoma and adenocarcinoma (**B**)

The median OS of all patients was 30.5 months (95% CI, not evaluable-not evaluable) (Fig. 2). The 12-month, 18-month, and 24-month OS rates were 95.7%, 77.1%, and 68.4%, respectively. Up to the last follow-up on August 3, 2022, 13 patients died, 32 were alive, and 4 were lost to follow-up.

A total of 43 patients had data for tumor response, and the ORR and DCR of patients were 25.6% and 97.7%, respectively. The ORR and DCR of patients with squamous cell carcinoma were 37.9% and 100%, respectively. The DCR of patients with adenocarcinoma was 92.9% (Table 4).

**Table 4:** Tumor responses

Response	All	Squamous cell carcinoma	Adenocarcinoma
N (missing)	43 (6)	29 (5)	14 (1)
BOR, n (%)			
CR	0	0	0
PR	11 (25.6%)	11 (37.9%)	0
SD	31 (72.1%)	18 (62.1%)	13 (92.9%)
PD	1 (2.3%)	0	1 (7.1%)
ORR, n (%)	11 (25.6%)	11 (37.9%)	0
DCR, n (%)	42 (97.7%)	29 (100.0%)	13 (92.9%)

BOR, best objective response; CR, complete response; PR, partial response; SD, stable disease; PD, progressive disease; ORR, objective response rate; DCR, disease control rate

### Safety

Treatment-related AEs of any grade were observed in 25 of 49 patients (51.0%). The most common treatment-related AEs were myelosuppression (14.3%), fever (10.2%), pneumonia (8.2%), abnormal liver function (8.2%), and telangiectasia (8.2%). AEs of grade 3 or worse were observed in nine patients (18.4%). The most common AE of grade 3 or worse was myelosuppression (grade 3, 6.1%; grade 4, 4.1%) (Table 5).

**Table 5:** Treatment-related adverse events

Events, n (%)	Any grade	≥ Grade3
Myelosuppression	7 (14.3%)	5 (10.2%)
Fever	5 (10.2%)	0
Pneumonia	4 (8.2%)	0
Abnormal liver function	4 (8.2%)	1 (2.0%)
Telangiectasia	4 (8.2%)	0
Hypothyroidism	3 (6.1%)	0
Fatigue	2 (4.1%)	0
Hyperuricemia	2 (4.1%)	2 (4.1%)
Anemia	2 (4.1%)	0
Myocarditis	2 (4.1%)	0
Enteritis	1 (2.0%)	0
Impaired pituitary function	1 (2.0%)	0
Hypokalemia	1 (2.0%)	0
Thrombocytopenia	1 (2.0%)	1 (2.0%)
Febrile neutropenia	1 (2.0%)	1 (2.0%)
Diarrhea	1 (2.0%)	0
Hyperglycemia	1 (2.0%)	0
Elevated troponin T	1 (2.0%)	0
Thyroiditis	1 (2.0%)	0
Nausea	1 (2.0%)	0
Loss of appetite	1 (2.0%)	0
Vomiting	1 (2.0%)	0
Rash/itching	1 (2.0%)	0
Allergic reaction	1 (2.0%)	0

A total of 12 patients (24.5%) had immune-related AEs. The most common immune-related AEs were pneumonia (8.2%) and telangiectasia (8.2%) (Table 6).

**Table 6:** Immune-related adverse events

Events, n (%)	All (*n* = 49)
Pneumonia	4 (8.2%)
Telangiectasia	4 (8.2%)
Hypothyroidism	1 (2.0%)
Hyperuricemia	2 (4.1%)
Myocarditis	2 (4.1%)
Enteritis	1 (2.0%)
Impaired pituitary function	1 (2.0%)
Hypokalemia	1 (2.0%)
Hyperglycemia	1 (2.0%)
Thyroiditis	1 (2.0%)
Rash/itching	1 (2.0%)

## Discussion

The efficacy of paclitaxel liposome combined with platinum in the first-line treatment of lung squamous cell carcinoma is well-documented [24]. Nevertheless, the combination of paclitaxel liposome and immunotherapy has not been evaluated in advanced NSCLC. We performed a retrospective, real-world study of 49 patients with advanced NSCLC who received paclitaxel liposome based chemotherapy plus PD-1/PD-L1 inhibitor, with 67.3% of the patients receiving the combined regimen as first-line treatment. Paclitaxel liposome based chemotherapy combined with PD-1/PD-L1 inhibitor showed effectiveness, with median PFS and OS in all patients of 9.7 months and 30.5 months, respectively, and an acceptable safety profile.

In a randomized controlled trial conducted by Zhang et al. that recruited patients with locally advanced or metastatic lung squamous cell carcinoma, paclitaxel liposome plus cisplatin had similar efficacy and better safety profile than gemcitabine plus cisplatin [24]. A previous study also reported that the combination of paclitaxel liposome, carboplatin, and concurrent radiotherapy is effective and safe in locally advanced squamous cell lung cancer [25]. The efficacy and safety of biweekly paclitaxel liposome plus nedaplatin for advanced squamous cell lung cancer has also been shown in a phase II trial [26]. Nevertheless, current studies mainly focused on patients with squamous cancer, and evidence demonstrating the effect of paclitaxel liposome in the treatment of lung adenocarcinoma is limited. According to the NCCN guidelines (version 5.2023) [17], pemetrexed-based immunotherapy is usually used as the preferred treatment in lung adenocarcinoma, but paclitaxel-based therapies such as carboplatin + albumin-bound paclitaxel + atezolizumab and carboplatin + paclitaxel + bevacizumab + atezolizumab are also recommended in the “other recommended” category. According to the Chinese Society of Clinical Oncology (CSCO) guidelines, carboplatin/cisplatinum + paclitaxel/paclitaxel liposome is recommended (2a) for advanced non-squamous NSCLC patients without driver gene mutations [27]. In this study, patients with squamous cell carcinoma and adenocarcinoma had median PFS of 11 months and 9.3 months, respectively. The results suggested that paclitaxel liposome based chemotherapy combined with immunotherapy had a satisfactory therapeutic effect either in squamous cell cancer or adenocarcinoma.

Most patients in this study received paclitaxel liposome based chemotherapy plus immunotherapy as first-line treatment, and the median PFS was 9.9 months. NSCLC cases without driver gene mutations have traditionally been treated with chemotherapy in the first-line setting. Nevertheless, the related benefit was limited, with a median PFS of about 5 months and a median OS of about 1 year [5]. Currently, several studies have demonstrated a survival benefit for ICIs combined with chemotherapy in the first-line treatment of patients with advanced NSCLC, including squamous cell carcinoma and adenocarcinoma [28]. Previous studies reported median PFS for patients administered ICI plus chemotherapy ranging from 5.5 months to 8.5 months and median OS ranging from 14.2 months to 17.2 months in the first-line treatment of non-squamous NSCLC [15, 16, 29–33]. In patients with squamous NSCLC administered ICI plus chemotherapy as first-line treatment, median PFS ranged from 4.2 months to 8.2 months, and median OS from 14.4 months to 29.2 months [8–12]. In this study, the median PFS was 11 months and 9.3 months in patients with squamous cell carcinoma and adenocarcinoma who received paclitaxel liposome based chemotherapy plus PD-1/PD-L1 inhibitor as first-line therapy, respectively. These results suggested that paclitaxel liposome based chemotherapy combined with immunotherapy as first-line treatment has a satisfactory therapeutic effect.

In this study, the median PFS was 7.3 months in patients who received paclitaxel liposome based chemotherapy plus immunotherapy in the second- or later-line setting, indicating that the paclitaxel liposome-based regimen was also effective as second-line or later-line treatment.

There were 16 patients who received maintenance therapy in this study, and five among them received paclitaxel liposome combined with immunotherapy, which may contribute to the overall prolongation of PFS and OS. A previous study also indicated that maintenance therapy after first-line treatment could improve the prognosis for patients with advanced NSCLC [34].

Despite the survival benefit of paclitaxel liposome based chemotherapy plus immunotherapy, the response rate in this study was relatively unsatisfactory. The ORRs of all patients and patients with squamous cell carcinoma were 25.6% and 37.9%, respectively. Previous findings have shown that the ORRs of ICIs plus chemotherapy were 45–75% for non-squamous NSCLC and 26-46% for squamous NSCLC in the first-line setting [28]. This might be associated with the inclusion of patients with second- or later-line treatment in our study, which possibly lowered the overall ORR. Furthermore, the lower response rate might also be related to the low dose of paclitaxel liposome used in this study. The present study had a median dose of 135 mg/m^2^ for paclitaxel liposome, while the recommended dose in the instructions is 135–175 mg/m^2^. The average age of patients in this study was older than that reported in previous clinical trials. Furthermore, one-third of the patients had previous systemic therapy. These factors might explain why clinicians generally choose a lower dose. The number of cycles of paclitaxel liposome in this study was similar to that reported by clinical trials [24]. Further study is needed to determine the optimal dose of paclitaxel liposome in combination therapy.

Regarding safety profile, paclitaxel liposome based chemotherapy combined with immunotherapy is well tolerated. A previous study showed that treatment with paclitaxel liposome plus cisplatin resulted in lower incidence of AEs, leading to treatment suspension or treatment discontinuation compared with gemcitabine plus cisplatin [24]. In this study, 51.0% of patients had all-grade AEs, and 18.4% had AEs of grade 3 or worse, which was lower than those of previous studies [8, 14, 15]. It might result from the lower dose of paclitaxel liposome and data bias due to the retrospective design of this study. Of the AEs observed, the most common was myelosuppression (14.3%), corroborating a previous study [24]. Besides, immune-related AEs were observed in 12 patients (24.5%), with pneumonia and telangiectasia being the most common.

There were some limitations in this study. First, biases were inevitable due to the retrospective nature of this study. Secondly, the sample size was relatively limited. Thirdly, this study lacked a control group. Further large-scale prospective clinical trials are warranted to confirm these results.

## Conclusions

In conclusion, paclitaxel liposome based chemotherapy combined with PD-1/PD-L1 inhibitor showed effectiveness in patients with advanced NSCLC, including both squamous cell carcinoma and adenocarcinoma, with acceptable safety profile.

## Data Availability

The datasets used and/or analyzed during the current study are available from the corresponding author upon reasonable request.

## Abbreviations

AEs: adverse events
CIs: confidence intervals
CR: complete response
DCR: disease control rate
ECOG PS: Eastern Cooperative Oncology Group performance status
ICIs: immune checkpoint inhibitors
IQR: interquartile range
NSCLC: non-small cell lung cancer
ORR: objective response rate
OS: overall survival
PR: partial response
RECIST: Response Evaluation Criteria in Solid Tumors
SD: stable disease
